# Supplementary material for: RprR is a plant-responsive regulator of exopolysaccharide production, biofilm formation, and virulence in Ralstonia pseudosolanacearum
Source: mBio. 2025 Dec 8;17(1):e02912-25. doi: 10.1128/mbio.02912-25 (PMC12802281; doi:10.1128/mbio.02912-25)
Supplement: Supplemental Figures — Figures S1 to S4 and supplemental methods and reference. [file mbio.02912-25-s0001.pdf]

Supplemental Figures and Methods

RprR is a plant-responsive regulator of EPS production, biofilm formation, and virulence in *Ralstonia pseudosolanacearum*

Bridget S. O'Banion*, Mariama D. Carter*, Jose A. Sanchez-Gallego, Hanlei Li, Nicholas J. Wagner, Lan Thanh Chu, Loan Bui, Tuan Minh Tran, and Caitilyn Allen

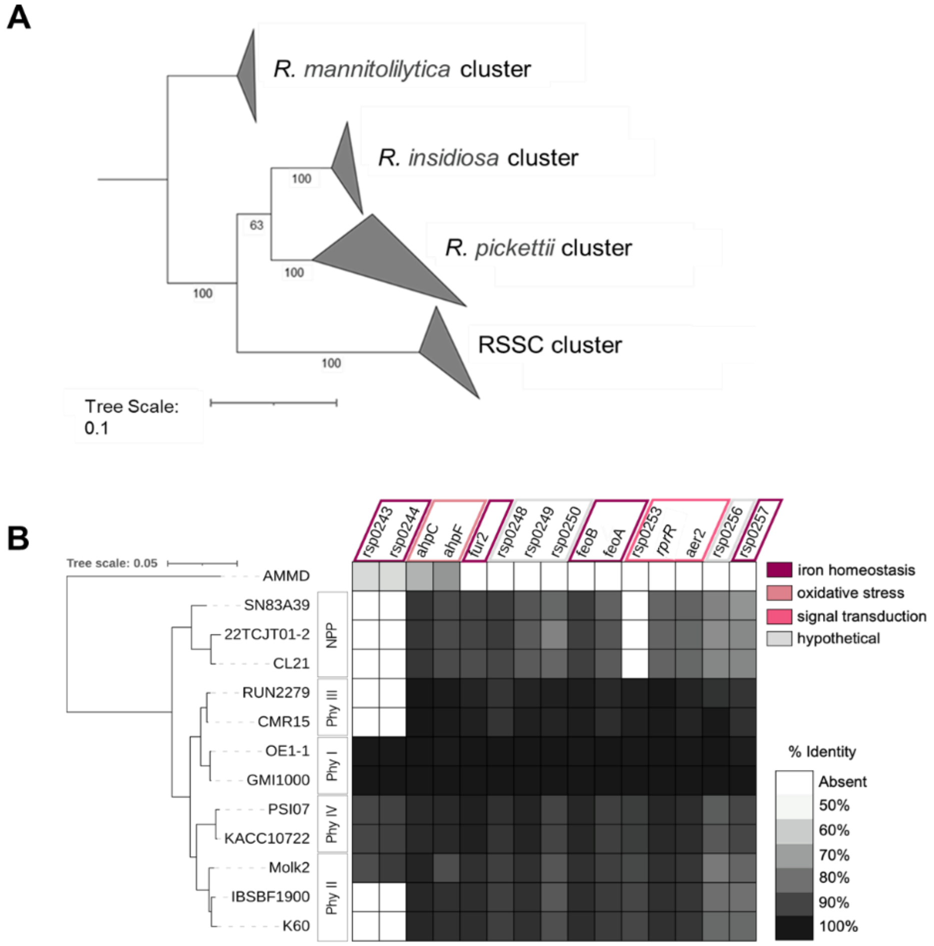

Supplemental Figure S1: The *rprR* gene is conserved across the genus *Ralstonia*, along with a 12-gene syntenic cluster. A) Maximum-likelihood gene tree constructed using the full-length *rprR* sequence from 106 *Ralstonia* genomes (See also Table S1 and supplementary methods). Individual branches were collapsed into 4 major taxonomic clusters. Bootstrap values are indicated on each node. Tree visualization was done in iTOL (1). **B)** Conservation of genes surrounding *rprR* in GMI1000. The protein blast function in KBase (2) was used to determine the conservation (≥50% protein sequence identity) of genes up- and downstream of *rprR* in GMI1000, indicated by degree of shading in the box representing each gene. The tree was edited in iTOL to overlay *rprR* conservation metadata and gene descriptions. Colored boxes outline genes based on the functional categories provided in the legend and mirror those shown in Figure 1A. AMMD = *Burkholderia ambifaria* AMMD (outgroup).

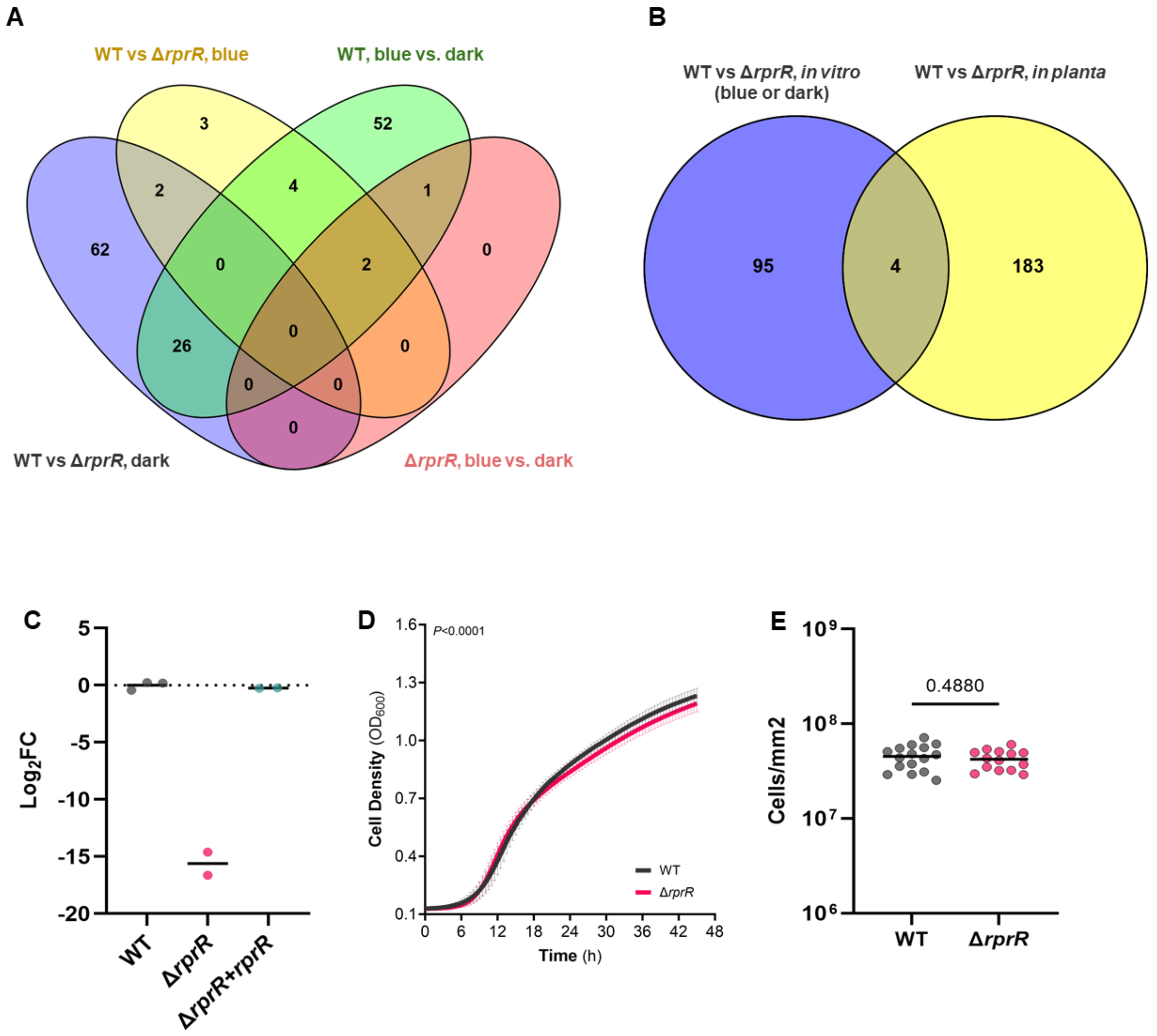

Supplemental Figure S2: Few DEGs overlap between treatments, expression of *rprR* is restored in the complemented mutant strain, and RprR does not affect colony cell density on dilute rich media. A-B) Venn diagrams showing the shared differentially expressed genes (DEGs) between treatments. Panel A shows the overlap between the four *in vitro* comparisons (WT or Δ*rprR* in blue light or dark). Panel B shows the overlap between DEGs *in vitro* (WT vs Δ*rprR*, blue or dark) and *in planta* (WT vs Δ*rprR*). See also Tables S3 and S4. Diagrams were made using Venny (v2.1.0, https://bioinfogp.cnb.csic.es/tools/venny/index.html). **C)** Expression of *rprR in vitro*. Total RNA was extracted from cell pellets and qRT-PCR was used to quantify *rprR* expression. Data is shown on a base-2 logarithmic scale as fold-change expression relative to wild-type *Rps* strain GMI1000 (WT); the black dashed line indicates value '1' (=mean WT expression). Each circle represents a biologically distinct culture (n=2-3 per strain). **D)** Growth of *Rps* strains in CPG broth at 28°C. Error bars represent SD. Data represents five independent experiments, each with 8 technical replicates (n=40 per strain, pvalue by two-way ANOVA). **E)** *Rps* colony cell density on dilute CPG media plates. Each circle represents data from a single agar-grown colony. Horizontal lines show the mean. The experiment was repeated three times with 2-6 colonies each (n=14-16 per treatment, p-value by Welch's T-test).

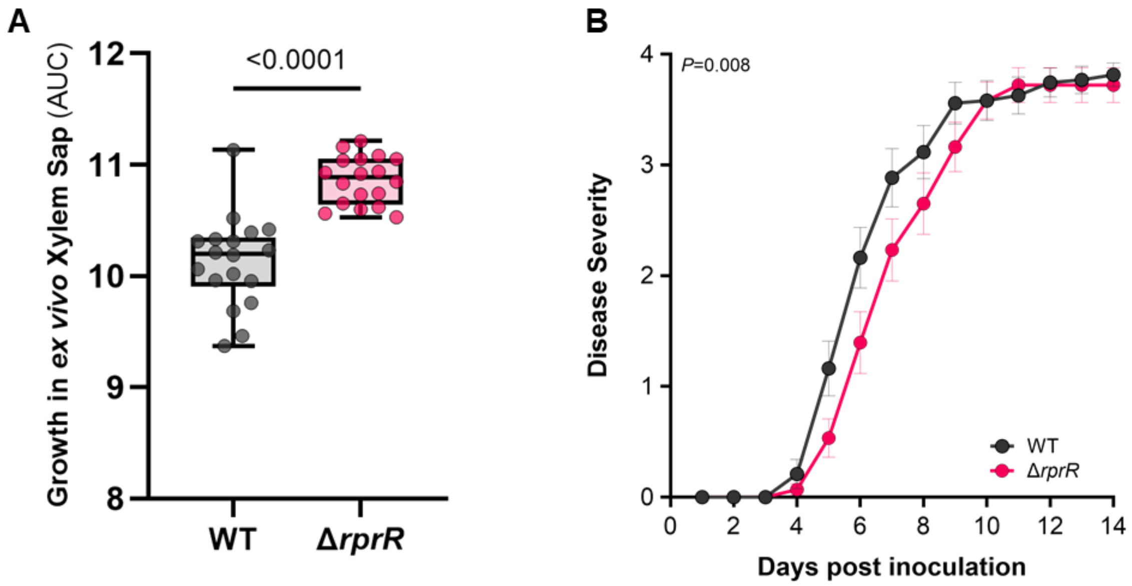

Supplemental Figure S3: The *Rps* Δ*rprR* mutant has a growth advantage in *ex vivo* tomato xylem sap, but a virulence defect following a naturalistic soil-soak infection of a susceptible host. A) Growth of *Rps* strain GMI1000 (WT) and Δ*rprR* mutant in *ex vivo* xylem sap. Each circle represents the area under the curve (AUC) calculated for an individual microtiter well. Whiskers represent min to max values. Data shown represent 3 independent experiments, each with 6 technical replicates (n=18 per treatment, p value by Welch's T-test). **B)** Wilt disease progress following soil-soak inoculation of unwounded wilt-susceptible Bonny Best tomato plants. Each symbol shows the mean disease index across three independent experiments, each containing 13-15 plants per treatment (n=43 per treatment, p-value = 0.008 by Repeated Measures two-way ANOVA). Error bars represent SEM.

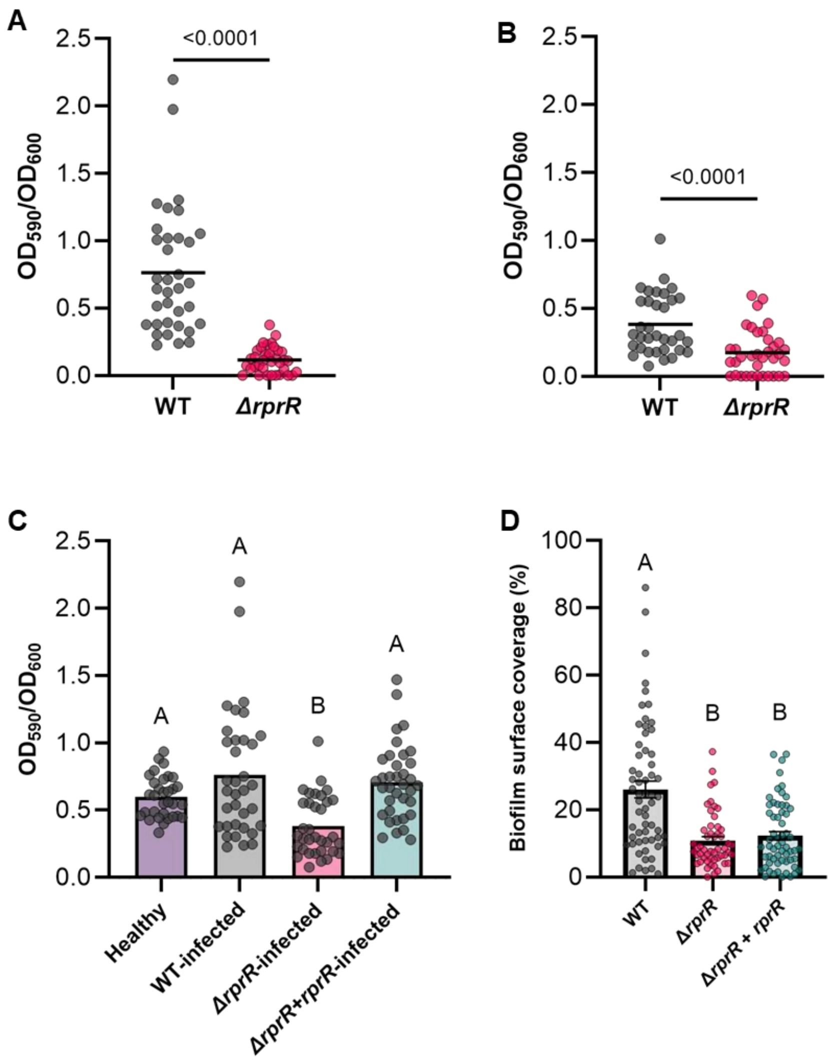

Supplemental Figure S4: Infected tomato stem homogenate supports *Rps* biofilm formation *in vitro*, and the Δ*rprR*+*rprR* strain did not complement biofilm formation under flow. A-B) *Rps* biofilm formation in filter-sterilized stem homogenate from infected tomato plants, measured by PVC platecrystal violet assay. Tomato plants were infected three days prior to stem collection with either wild-type *Rps* GMI1000 (WT) (A) or Δ*rprR* (B). Each circle represents biofilm data from a single microtiter well. Lines indicate the mean. Data reflects three independent experiments with 12 replicates each (n=36 per treatment). Outliers identified by a ROUT analysis (default parameters, implemented in GraphPad Prism) were removed prior to analysis and visualization. Data was analyzed using a Mann-Whitney test (A) and Welch's T-test (B). **C)** Biofilm formation of *Rps* GMI1000 wild-type in filter-sterilized stem homogenate from healthy and infected tomato plants. Tomato plants were infected three days prior to stem collection with either WT, Δ*rprR*, or Δ*rprR*+*rprR*, or left un-inoculated (Healthy). Each circle represents biofilm data from a single microtiter well. Lines indicate the mean. Data reflect three independent experiments with 12 replicates each (n=36 per treatment). Outliers identified by a ROUT analysis (default parameters, implemented in GraphPad Prism) were removed prior to analysis and visualization. Different letters indicate differences between groups (Lognormal Brown Forsythe and Welch ANOVA). Data for healthy and WT-infected treatments are also shown in Figure 4C and S4A. **D)** Biofilm formation in *ex vivo* xylem

sap under continuous flow (see Fig. 4E). Each circle represents the crystal violet stain coverage % at a single location within a single channel. The experiment was repeated twice, with 10 channels imaged for each device, at three different locations along each channel (n=60 per treatment). Different letters indicate differences between groups (determined by Kruskal-Wallis and Dunn's multiple comparisons tests). The WT and Δ*rprR* data shown in this figure is also shown in figure 4D. Outliers identified by a ROUT analysis (default parameters, implemented in GraphPad Prism) were removed prior to analysis and visualization.

Supplemental Methods

***In silico* analyses of RprR.** *Ralstonia* genomes were obtained from the Joint Genome Institute's Integrated Microbial Genomes webserver (JGI IMG) in October 2023 (5). All genomes listed under the genus *Ralstonia* were manually curated to remove duplicate entries and assemblies with >60 scaffolds (final n = 111, Table S1). A "cassette search" of the genome set was performed using default parameters and the following hooks: pfam00672 (HAMP), pfam08447 (PAS_3), pfam13426 (PAS_9), pfam00990 (GGDEF), and pfam00563 (EAL). The hooks represent the JGI annotated pfam functions of the *Rps* GMI1000 *rprR* gene, excluding the CACHE domain, which we found was not consistently annotated within *rprR* across the genus. This functional search identified a single hit (hit = all hooks present within a 4kb genomic region) within 106 genomes. The amino acid sequence of the identified *rprR* genes (n = 106) were downloaded from JGI and subsequently uploaded to the CIPRES gateway for alignment using MUSCLE (default parameters, v3.7) (6, 7). The multiple-sequence alignment (MSA) was manually curated in Jalview (v2.11.2.7) (8) before building a maximum-likelihood tree in the CIPRES gateway (RAxML-HPC BlackBox, v8.2.12) (6, 9). The tree was uploaded to iTOL for visualization and formatting (1).

To visualize genomic context in Figure S1B, a phylogenetic tree was constructed on KBase (2) with 9 RSSC strains (representing all 4 phylotypes), 3 non-plant pathogenic (NPP) *Ralstonia* spp., and *Burkholderia ambifaria* AMMD as an outgroup. The phylogenetic analysis is based on 49 conserved genes (10). The protein blast function in KBase was used to determine the conservation (≥50% protein sequence identity) of genes up- and downstream of *rprR* in GMI1000.

qRT-PCR. To compare *rprR* gene expression across GMI1000 wild-type, Δ*rprR*, and Δ*rprR* + *rprR*, cultures were grown overnight in CPG to an OD_{600} 0.2-0.6 (log phase) before pelleting via centrifugation. Total RNA was isolated from the cell pellets using a Direct-zol RNA extraction kit (Zymo Research). RNA was reverse transcribed into cDNA with the SuperScript VILO cDNA Synthesis Kit (Invitrogen). A targeted qRT-PCR measured expression of *rprR* and two normalization genes (*rplM* and *serC*) using primers listed in Table S5. The ΔΔCt method was used to calculate relative gene expression in Δ*rprR* and Δ*rprR* + *rprR* compared to wild-type.

Agar-grown colony cell density. Strains were prepared as described in the main methods and resuspended to a concentration of approximately 50-100 CFUs/mL in water. A 100 uL volume of each suspension was spread on an agar plate containing a modified dilute rich media (2 g/L peptone, 0.2 g/L casamino acids, 1.8 g/L glucose, 0.2 g/L yeast extract) and incubated in the dark at 28°C. After 4 days, the plates were imaged and two orthogonal measurements of each colony diameter were taken and averaged. This measurement was used to calculate the area of the colony ($A = \pi r^2$). The measured colonies were then cored from the plate using the top end of a 1,000 µL pipet tip, homogenized in water, and serially diluted.

Growth in *ex vivo* xylem sap. Xylem sap was collected as described in the main methods. Cultures were prepared as described in the main methods and resuspended to a final OD_{600} of 0.01 in thawed xylem sap. Cell suspensions were aliquoted into flat-bottomed 96-well plates (200 µL/well, Corning Costar Ref# 3370) and incubated at 28°C with slow continuous shaking in a Biotek plate reader. The OD_{600} was measured every 30 minutes for 24 hours.

Supplemental References

1. Letunic I, Bork P. 2021. Interactive Tree Of Life (iTOL) v5: an online tool for phylogenetic tree display and annotation. Nucleic Acids Res 49:W293–W296.

2. Arkin AP, Cottingham RW, Henry CS, Harris NL, Stevens RL, Maslov S, Dehal P, Ware D, Perez F, Canon S, Sneddon MW, Henderson ML, Riehl WJ, Murphy-Olson D, Chan SY, Kamimura RT, Kumari S, Drake MM, Brettin TS, Glass EM, Chivian D, Gunter D, Weston DJ, Allen BH, Baumohl J, Best AA, Bowen B, Brenner SE, Bun CC, Chandonia J-M, Chia J-M, Colasanti R, Conrad N, Davis JJ, Davison BH, DeJongh M, Devoid S, Dietrich E, Dubchak I, Edirisinghe JN, Fang G, Faria JP, Frybarger PM, Gerlach W, Gerstein M, Greiner A, Gurtowski J, Haun HL, He F, Jain R, Joachimiak MP, Keegan KP, Kondo S, Kumar V, Land ML, Meyer F, Mills M, Novichkov PS, Oh T, Olsen GJ, Olson R, Parrello B, Pasternak S, Pearson E, Poon SS, Price GA, Ramakrishnan S, Ranjan P, Ronald PC, Schatz MC, Seaver SMD, Shukla M, Sutormin RA, Syed MH, Thomason J, Tintle NL, Wang D, Xia F, Yoo H, Yoo S, Yu D. 2018. KBase: The United States Department of Energy systems biology knowledgebase. Nat Biotechnol 36:566–569.

3. Goujon M, McWilliam H, Li W, Valentin F, Squizzato S, Paern J, Lopez R. 2010. A new bioinformatics analysis tools framework at EMBL-EBI. Nucleic Acids Res 38:W695–9.

4. Sievers F, Wilm A, Dineen D, Gibson TJ, Karplus K, Li W, Lopez R, McWilliam H, Remmert M, Söding J, Thompson JD, Higgins DG. 2011. Fast, scalable generation of high quality protein multiple sequence alignments using Clustal Omega. Mol Syst Biol 7:539.

5. Chen I-MA, Chu K, Palaniappan K, Ratner A, Huang J, Huntemann M, Hajek P, Ritter SJ, Webb C, Wu D, Varghese NJ, Reddy TBK, Mukherjee S, Ovchinnikova G, Nolan M, Seshadri R, Roux S, Visel A, Woyke T, Eloe-Fadrosh EA, Kyrpides NC, Ivanova NN. 2022. The IMG/M data management and analysis system v.7: content updates and new features. Nucleic Acids Res https://doi.org/10.1093/nar/gkac976.

6. Miller MA, Pfeiffer W, Schwartz T. 2010. Creating the CIPRES Science Gateway for inference of large phylogenetic trees, p. 1–8. *In* 2010 Gateway Computing Environments Workshop (GCE).

7. Edgar RC. 2004. MUSCLE: multiple sequence alignment with high accuracy and high throughput. Nucleic Acids Res 32:1792–1797.

8. Waterhouse AM, Procter JB, Martin DMA, Clamp M, Barton GJ. 2009. Jalview Version 2-- a multiple sequence alignment editor and analysis workbench. Bioinformatics 25:1189– 1191.

9. Stamatakis A. 2014. RAxML version 8: a tool for phylogenetic analysis and post-analysis of large phylogenies. Bioinformatics 30:1312–1313.

10. Lowe-Power T, Avalos J, Bai Y, Munoz MC, Chipman K, Tom CE, Williams D. 2024. A metaanalysis of the known global distribution and host range of the *Ralstonia* species complex. bioRxiv doi: https://doi.org/10.1101/2020.07.13.189936

11. Boucher C, Martinel A, Barberis P, Alloing G, Zischek C. 1986. Virulence genes are carried by a megaplasmid of the plant pathogen *Pseudomonas solanacearum*. Mol Gen Genet 205:270–275.

12. Castañeda A, Reddy JD, El-Yacoubi B, Gabriel DW. 2005. Mutagenesis of all eight *avr* genes in *Xanthomonas campestris* pv. campestris had no detected effect on pathogenicity, but one *avr* gene affected race specificity. Mol Plant Microbe Interact 18:1306–1317.

13. Monteiro F, Solé M, van Dijk I, Valls M. 2012. A chromosomal insertion toolbox for promoter probing, mutant complementation, and pathogenicity studies in *Ralstonia solanacearum*. Mol Plant Microbe Interact 25:557–568.

14. Khokhani D, Lowe-Power TM, Tran TM, Allen C. 2017. A Single Regulator Mediates Strategic Switching between Attachment/Spread and Growth/Virulence in the Plant Pathogen *Ralstonia solanacearum*. mBio 8:00895-17.
